# Supplementary material for: Super-resolution imaging uncovers the nanoscopic segregation of polarity proteins in epithelia
Source: eLife. 2022 Nov 7;11:e62087. doi: 10.7554/eLife.62087 (PMC9674336; doi:10.7554/eLife.62087)
Supplement: Figure 1—source data 1. [file elife-62087-fig1-data1.docx]

Figure 1-source data 1

The number of junctions in each replicate is given between commas:
Pl: planar, AB: apico-basal

| Label  Sample | PAR3 Occl | | aPKC Occl | | PAR6β Occl | | PATJ Occl | | CRB3A ZO-1 | |
| --- | --- | --- | --- | --- | --- | --- | --- | --- | --- | --- |
|  | Pl | AB | Pl | AB | Pl | AB | Pl | AB | Pl | AB |
| Human  junctions | (22,34,24) | (9,6,7) | (13,15,16) | (7,9,10) | (10,8,9) | (6,7,8) | (47,12,9) | (16,12,11) | (4,8,13) | (6,6,10) |

| Label  Sample | PALS1 ZO-1 | | E-cad ZO-1 |
| --- | --- | --- | --- |
|  | Pl | AB | AB |
| Human  junctions | (9,23,22) | (4,8,8) | (13,10,9) |

| Label  Sample | PAR3 ZO-1 | | PALS1 ZO-1 | | E-cad ZO-1 |
| --- | --- | --- | --- | --- | --- |
|  | Pl | AB | Pl | AB | AB |
| Mouse  junctions | (7,16,18) | (6,14,11) | (31,38,9) | (11,11,5) | (9,18,9) |
