# Supplementary material for: Super-resolution imaging uncovers the nanoscopic segregation of polarity proteins in epithelia
Source: eLife. 2022 Nov 7;11:e62087. doi: 10.7554/eLife.62087 (PMC9674336; doi:10.7554/eLife.62087)
Supplement: Figure 1—figure supplement 1—source data 1. [file elife-62087-fig1-figsupp1-data1.docx]

Figure 1-figure supplement 1-source data 1

The number of junctions in each replicate is given between commas:
Pl: planar, AB: apico-basal

| Label  Sample | ZO-1 Occl | |
| --- | --- | --- |
|  | Pl | AB |
| Human  junctions | (6,8,9) | (12,3,17) |
