# Supplementary material for: Super-resolution imaging uncovers the nanoscopic segregation of polarity proteins in epithelia
Source: eLife. 2022 Nov 7;11:e62087. doi: 10.7554/eLife.62087 (PMC9674336; doi:10.7554/eLife.62087)
Supplement: Figure 2—source data 1. [file elife-62087-fig2-data1.docx]

Figure 2-source data 1

The number of junctions in each replicate is given between commas:
Pl: planar, AB: apico-basal

| Label  Sample | PAR3 Occl | | aPKC Occl | | PAR6β Occl | | PATJ Occl | | CRB3A ZO-1 | |
| --- | --- | --- | --- | --- | --- | --- | --- | --- | --- | --- |
|  | Pl | AB | Pl | AB | Pl | AB | Pl | AB | Pl | AB |
| Caco-2  junctions | (30,13,16) | (8,7,6) | (16,4,9) | (9,6,6) | (18,17,21) | (9,11,6) | (33,14,15) | (8,7,8) | (5,12,14) | (11,7,10) |

| Label  Sample | PALS1 ZO-1 | | E-cad ZO-1 |
| --- | --- | --- | --- |
|  | Pl | AB | AB |
| Caco-2  junctions | (14,10,7) | (3,7,7) | (9,9,8) |
