# Supplementary material for: Super-resolution imaging uncovers the nanoscopic segregation of polarity proteins in epithelia
Source: eLife. 2022 Nov 7;11:e62087. doi: 10.7554/eLife.62087 (PMC9674336; doi:10.7554/eLife.62087)
Supplement: Figure 4—source data 1. [file elife-62087-fig4-data1.docx]

Figure 4-source data 1

The number of junctions in each replicate is given between commas:

| Label | PATJ  PALS1 | PATJ  aPKC | | PALS1 aPKC | PALS1 PAR6β | PAR6β aPKC | CRB3A PALS1 | CRB3A PATJ |
| --- | --- | --- | --- | --- | --- | --- | --- | --- |
| Number of junctions | (26,15,14) | (82,28,18) | | (56,14,17) | (41,17,13) | (25,17,20) | (20,33,13) | (32,34,13) |
| Label | CRB3A aPKC | CRB3A PAR6β | | CRB3A PAR3 | CRB3A  ZO-1 | PAR3 PALS1 | PAR3 aPKC | PAR3 OCLN |
| Number of junctions | (20,32,13) | (25,18,10) | | (10,8,6) | (16,8,9) | (8,10,9) | (20,33,13) | (7,7,7) |
| Label | PATJ-Alexa568 PATJ-Alexa532 | |  |  |  |  |  |  |
| Number of junctions | (15,9,8) | |  |  |  |  |  |  |
