# Supplementary material for: Super-resolution imaging uncovers the nanoscopic segregation of polarity proteins in epithelia
Source: eLife. 2022 Nov 7;11:e62087. doi: 10.7554/eLife.62087 (PMC9674336; doi:10.7554/eLife.62087)
Supplement: Figure 4—figure supplement 1—source data 1. [file elife-62087-fig4-figsupp1-data1.docx]

Figure 4-figure supplement 1-source data 1

The number of junctions for each experiment is given between commas (one cell culture replicate per experiment):

| Label | PATJ  PALS1 | PATJ  aPKC | PALS1 aPKC | PALS1 PAR6β | PAR6β aPKC | CRB3A PALS1 |
| --- | --- | --- | --- | --- | --- | --- |
| Number of junctions | 7 | 7 | 7 | 8 | 9 | 7 |
| Label | CRB3A aPKC | CRB3A PAR3 | CRB3A  ZO-1 | PAR3 PALS1 | PAR3 aPKC | PAR3 OCLN |
| Number of junctions | 8 | 36 | 10 | 19 | 10 | 8 |
