# Supplementary figures and images for: Super-resolution imaging uncovers the nanoscopic segregation of polarity proteins in epithelia
Source: eLife. 2022 Nov 7;11:e62087. doi: 10.7554/eLife.62087 (PMC9674336; doi:10.7554/eLife.62087)

$\alpha$ Tub

WT KI

Tub

WT KI

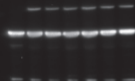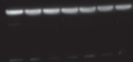

Supplement: Appendix 1—figure 1—source data 1. [file elife-62087-app1-fig1-data1.zip › Annotated - Blot 1 and 2 anti-Tub.pdf]

$\alpha$ PAR6 $\beta$

$\alpha$ GFP

WT KI

WT KI

PAR6 $\beta$ -Cit

PAR6 $\beta$ -Citrine

PAR6 $\beta$

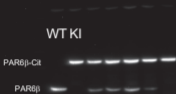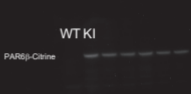

Supplement: Appendix 1—figure 1—source data 1. [file elife-62087-app1-fig1-data1.zip › Annotated - Blot 1 anti-PAR6beta blot 2 anti-GFP.pdf]

$\alpha$ Tub

Caco-2

siCT siCRB3

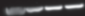

Supplement: Appendix 1—figure 1—source data 1. [file elife-62087-app1-fig1-data1.zip › Annotated - Blot 3 - anti-tubulin.pdf]

$\alpha$ CRB3

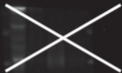

siCT siCRB3

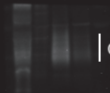

CRB3

Supplement: Appendix 1—figure 1—source data 1. [file elife-62087-app1-fig1-data1.zip › Annotated -Blot 3 (bottom) anti-CRB3.pdf]

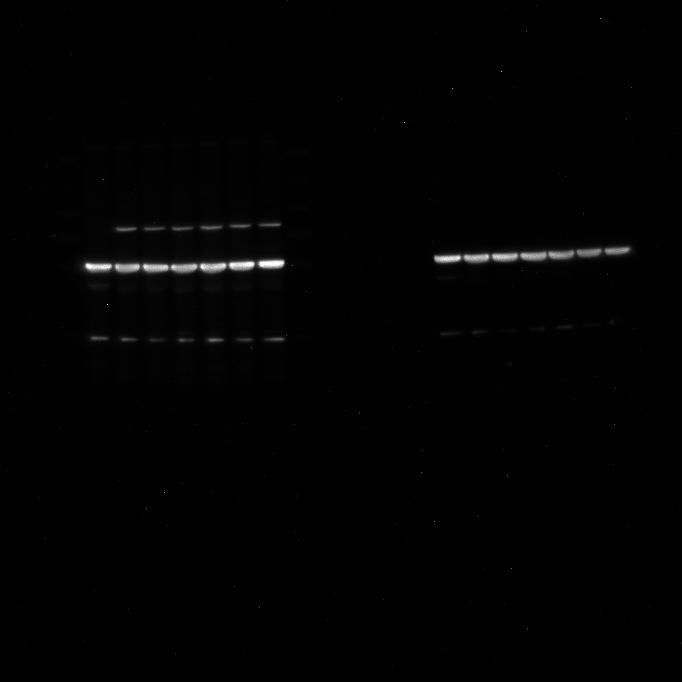

Supplement: Appendix 1—figure 1—source data 1. [file elife-62087-app1-fig1-data1.zip › Blot 1 and 2 anti-Tub.tif]

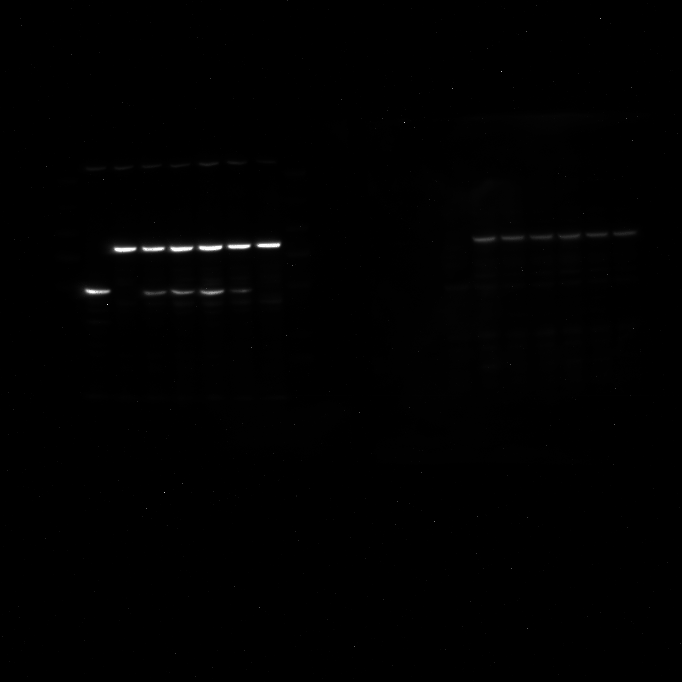

Supplement: Appendix 1—figure 1—source data 1. [file elife-62087-app1-fig1-data1.zip › Blot 1 anti-PAR6beta blot 2 anti-GFP.tif]

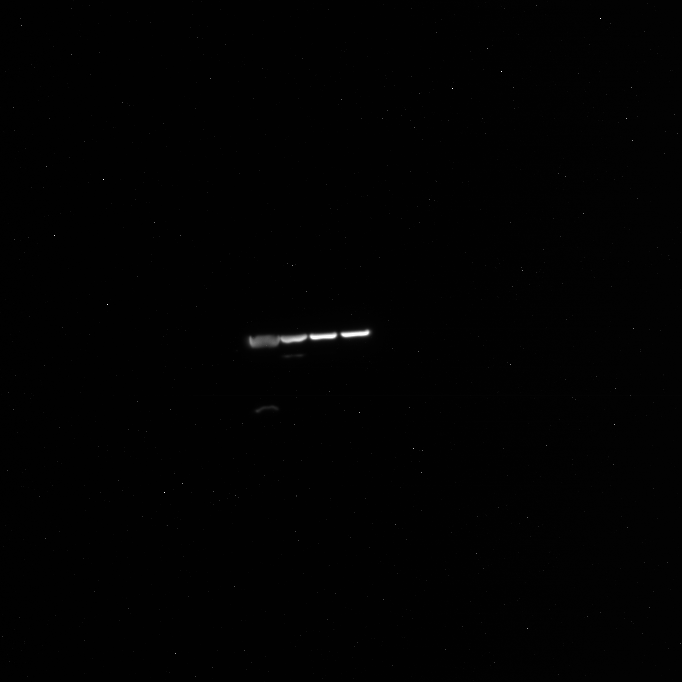

Supplement: Appendix 1—figure 1—source data 1. [file elife-62087-app1-fig1-data1.zip › Blot 3 - anti-tubulin.tif]

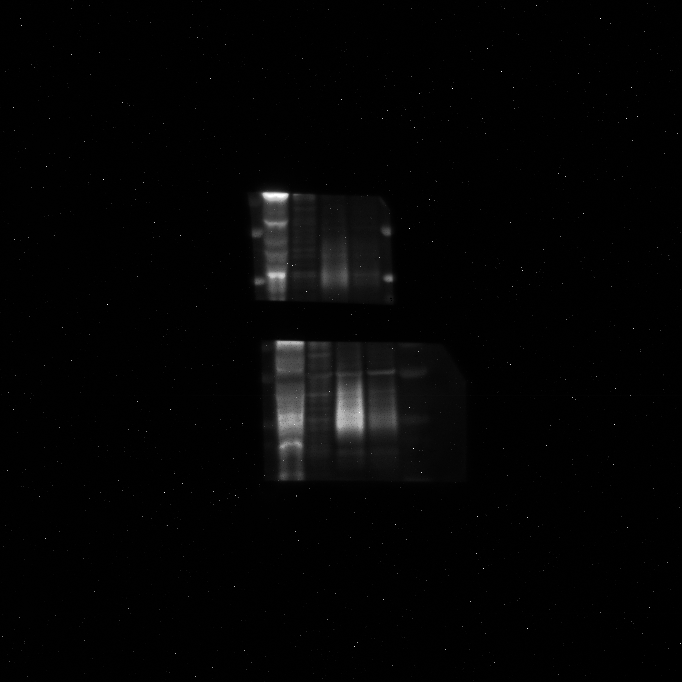

Supplement: Appendix 1—figure 1—source data 1. [file elife-62087-app1-fig1-data1.zip › Blot 3 (bottom) anti-CRB3.tif]

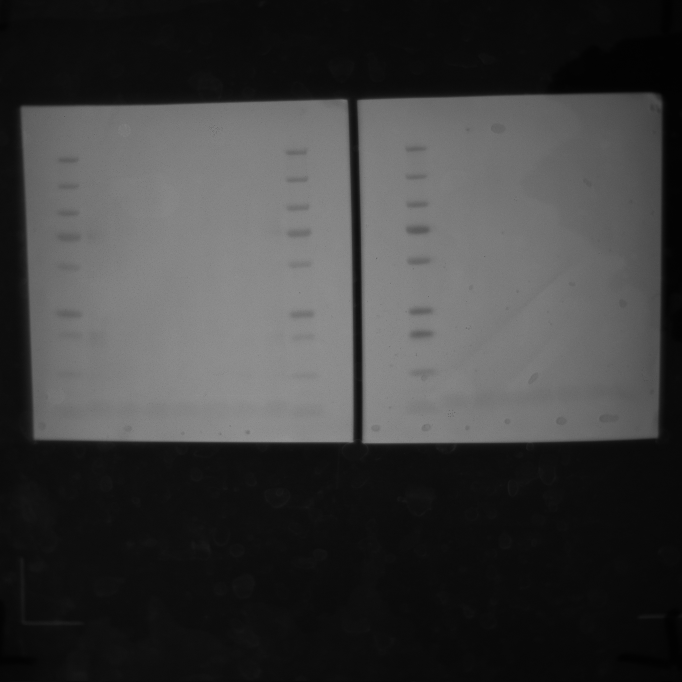

Supplement: Appendix 1—figure 1—source data 1. [file elife-62087-app1-fig1-data1.zip › white light - Blot 1 and 2 anti-Tub.tif]

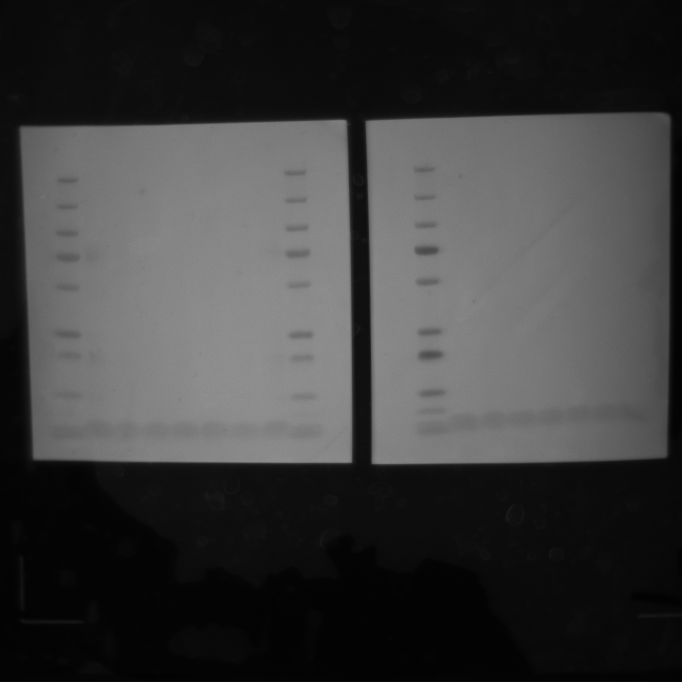

Supplement: Appendix 1—figure 1—source data 1. [file elife-62087-app1-fig1-data1.zip › white light - Blot 1 anti-PAR6beta blot 2 anti-GFP.tif]

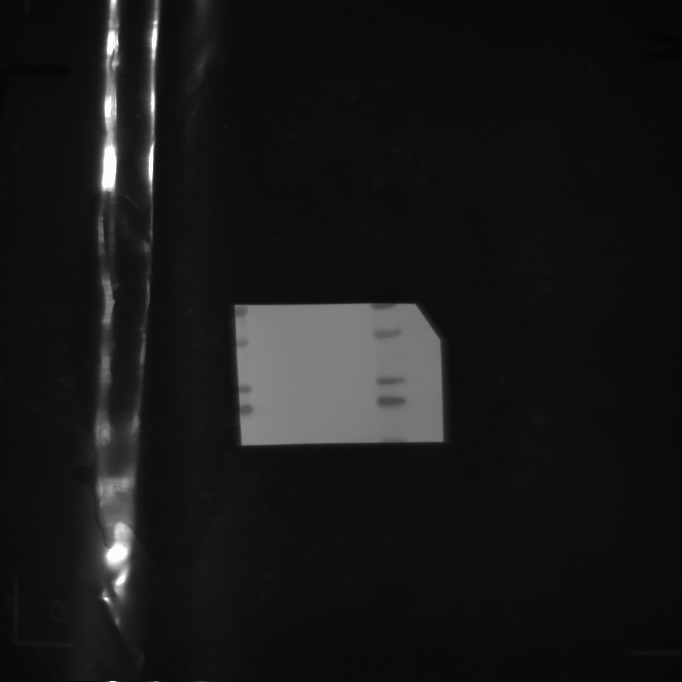

Supplement: Appendix 1—figure 1—source data 1. [file elife-62087-app1-fig1-data1.zip › white light - Blot 3 - anti-tubulin.tif]

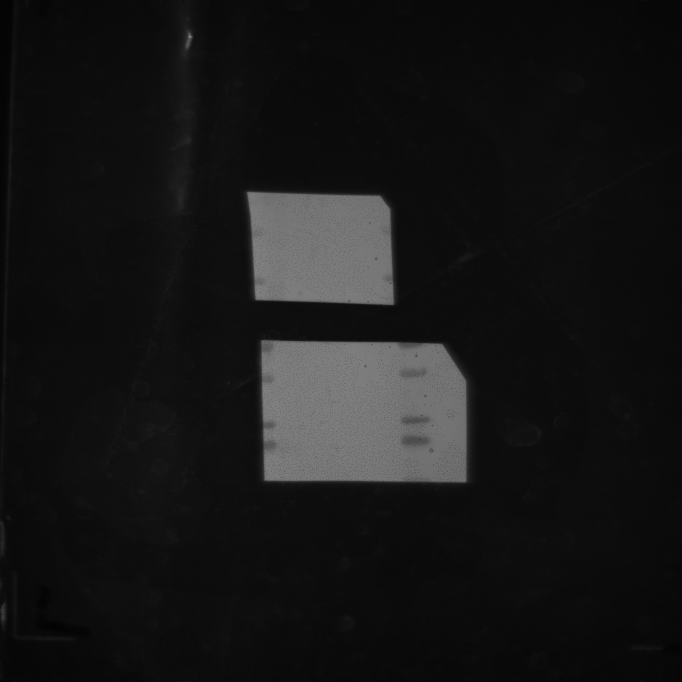

Supplement: Appendix 1—figure 1—source data 1. [file elife-62087-app1-fig1-data1.zip › white light - Blot 3 (bottom) anti-CRB3.tif]
